# Impact of SARS-CoV-2 on the mobility behaviour in Germany

**DOI:** 10.1186/s12544-021-00469-3

**Published:** 2021-01-26

**Authors:** Juliane Anke, Angela Francke, Lisa-Marie Schaefer, Tibor Petzoldt

**Affiliations:** Technische Universität Dresden, Traffic and Transportation Psychology, 01062 Dresden, Germany

**Keywords:** Mobility behaviour, Transport, Pandemic, COVID-19, SARS-CoV-2, Corona, Mode choice

## Abstract

**Background:**

The COVID-19 pandemic and the measures taken to combat it led to severe constraints for various areas of life, including mobility. To study the effects of this disruptive situation on the mobility behaviour of entire subgroups, and how they shape their mobility in reaction to the special circumstances, can help to better understand, how people react to external changes.

**Methodology:**

Aim of the study presented in this article was to investigate to what extent, how and in what areas mobility behaviour has changed during the outbreak of SARS-CoV-2 in Germany. In addition, a focus was put on the comparison of federal states with and without lockdown in order to investigate a possible contribution of this measure to changes in mobility. We asked respondents via an online survey about their trip purposes and trip frequency, their choice of transport mode and the reasons for choosing it in the context of the COVID-19 crisis. For the analyses presented in this paper, we used the data of 4157survey participants (2512 without lockdown, 1645 with lockdown).

**Results:**

The data confirmed a profound impact on the mobility behaviour with a shift away from public transport and increases in car usage, walking and cycling. Comparisons of federal states with and without lockdown revealed only isolated differences. It seems that, even if the lockdown had some minor effects, its role in the observed behavioural changes was minimal.

## Introduction

COVID-19 has reshaped the world as we know it. That much is clear, even if, at the time of this paper’s writing, the result of this transformation process is still impossible to foresee. Many lives have already been lost. Health care systems have been put under massive strain, and not all were able to cope. Economies have been hit hard, and livelihoods have been destroyed. And even with a vaccine available, many more casualties, directly or indirectly caused by COVID-19, are to be expected.

The effects on the transport system were severe as well. At its lowest point in April, EUROCONTROL [8] reported a 89% decrease in air traffic in Europe. Use of the London Underground went down by 96% of the typical demand [27]. San Francisco’s Bay Area Rapid Transit [4] saw a reduction in ridership of up to 94%. In Tokyo, where public transport accounts for more than half of all daily trips [16], Toei subways reported a maximum decline in ridership of 69% [26]. Vehicle miles travelled went down by 88% of the pre-COVID-19 level in Spain, and by more than 70% in many other European nations [20].

Although the COVID-19 pandemic constitutes a unique and, in its scale, unprecedented scenario, the pandemic’s effects on mobility behaviour are not surprising, as, from previous crises, there is knowledge on how societies might respond to threats to public health. Surveys conducted during outbreaks of SARS [17], A(H1N1), also known as “swine flu” [6, 15] and A(H7N9), aka “bird flu” [19], consistently found that respondents tended to avoid crowded places, and public transport in particular, as preventive measures. Others also found that respondents delayed or cancelled air travel plans, and anticipated to use public transport less frequently [12]. Similarly, other crises, such as terrorist attacks, have been found to affect travel behaviour and modal choice, typically at the expense of public transport [3, 11].

It should be noted, however, that many of the behavioural adaptations that occurred during previous crises were the result of individual decision making that aimed at avoiding some form of risk and overall, people were mostly free to move where and how they preferred. In contrast, COVID-19 was met with strict restrictions on individual freedoms all over the world, restrictions that massively affected everyday life, with direct and indirect implications also for mobility behaviour. Borders were closed, schools and day care facilities had to shut down, restaurants were not allowed to welcome guests, and only essential shops remained open. Many employees were encouraged to work from home, others were let go from their jobs because their employers were losing business as a result of the restrictions. Citizens were advised to adhere to social distancing protocols and wear face masks in public space. Many countries also introduced temporary lockdowns of different magnitude.

### Situation of COVID-19 in Germany

In Germany, COVID-19 arrived mid-February 2020, when returnees from Wuhan, China, were isolated. By the end of February, the Federal Ministry of Health and the Federal Ministry of the Interior, Building and Community set up a crisis response team. Cases of infection were confirmed in two federal states. Two days later, the crisis response team recommended the cancellation of major events. Border traffic was monitored, and regional and long-distance travellers with symptoms had to be reported to the health authorities. At the end of March, schools, day care facilities, playgrounds, non-essential shops, zoos, botanical gardens and hairdressers were ordered to stay closed. When shopping for essentials, Germans were required to keep a minimum distance of 1.5 m to others and use a face mask inside shops. In addition, six of the sixteen federal states introduced a temporary lockdown that lasted from four up to 7 weeks [25]. The lockdown meant a prohibition to leave the house without sound reason [25]. Among those sound reasons were the commute to work, grocery shopping, doctor’s appointments, important appointments (e.g. exams), and individual outdoor sports activities (e.g., cycling) or walks (within a certain radius of the home, depending on the federal state) [9].

As a consequence of the COVID-19 outbreak, flight passenger numbers decreased by up to 63% in March 2020 compared to March 2019 [23]. The number of rail passengers went down by roughly 40% for the same period. The number of people using public transport (short distance) decreased by 11% for the first quarter of 2020 (data not collected on monthly basis) [24]. According to Google [13], visitation of retail and recreation facilities went down by as much as 77% at the end of March compared to a reference day from earlier in 2020 (median of the same weekday from January 3rd to February 6th). Similar reductions were reported for transit stations with a decrease up to 68%. Workplace attendance fell by as much as 47% in early April. Analyses conducted by the Project Group Computational Epidemiology [21], who used mobile phone data to identify movements, showed that, at the end of March, weekday movements were reduced by as much as 38%. Similar results were reported by infas [10], who analysed tracking data of 1000 volunteers. The data also showed an increased proportion of walking and cycling, while the modal share of public transport decreased. Indeed, a survey conducted in early April [7] found that a considerable portion of the respondents would feel uncomfortable using public transport at time of questioning (57–63%, dependent on specific mode of transport). Six percent of those who did not own a car reported to consider buying one as result of the COVID-19 outbreak.

Aim of the analyses presented in this paper was to provide a more detailed understanding of the changes in individual mobility behaviour following the COVID-19 outbreak in Germany. Using survey data collected in the early stages of the crisis, the intent was to address questions of modal choice, trip purposes, and changes thereof. A specific focus was put on potential differences that might have arisen from the different lockdown regimes in the German federal states.^1^

## Method

### Survey

As the basis for our analyses, we used data that we acquired through a large-scale online survey on mobility behaviour. The survey was set up in direct response to the COVID-19 outbreak in Germany. It was launched on March 20th 2020 and distributed through multiple channels such as social media, newsletters and mailing lists. Overall, the survey consisted of three parts. In the first part, participants were required to provide information on their mobility behaviour before the outbreak of the virus. We asked them to report, e.g., their typical means of transport, their most frequent trip destinations and their trip purpose. The second part contained comparable questions about their current mobility behaviour, i.e. under the changed conditions since the beginning of the virus outbreak and the associated restrictions. The third part asked participants for a prediction of their mobility behaviour after the end of the pandemic. An overview of the three parts of the survey, the items it contained and their origin is shown in Table 1. By May 15th 2020, 6126 participants had completed the survey. A follow-up data collection with the same participants is scheduled for the same period in 2021 (21.03.-15.05.).

**Table 1:** Three parts of the survey, included items and item origin

Items regarding	Items	Item origin
Mobility before COVID-19 (Part 1)		
*Modal split, Choice of means of transport*	3	Self-developed
*Trip purposes, Main trip purpose, Distance main trip purpose,*	3	
*Identification based on means of transport*	1	Self-developed
Mobility during COVID-19 (Part 2)		
*Restrictions at the time of the survey*	3	Self-developed
*Changes in Modal split, Choice of means of transport, Trip purposes, Reasons for changes in mobility*	7	Self-developed
*Attitudes, Social norms (not subject of the present paper)*	10	Based on standard items (following Armitage [2]; see also Ajzen [1])
Mobility after COVID-19 & Sociodemographics (Part 3)		
*Predictions of long-term effects on traffic due to COVID-19*	1	Self-developed
*Age, Gender, Federal state, Education, Employment status,* etc.	11	Sosci-template [18]
*Mobility related characteristics (drivers’ licence ownership, subscription for public transport, cars and serviceable bicycles per household)*	4	Self-developed
*Infrastruktural premises at the place of residence regarding public transport, driving, cycling and walking*	1	Self-developed

### Sample

For the analyses presented in this paper, only responses that were recorded from March 21st to April 19th were included. This was necessary to ensure that the data only covered the period of the lockdown in those six federal states that had instated one (the Free State of Saxony ended its lockdown on April 20th, others upheld this restriction up until May 9th). To be included in the analysis, participants had to be over the age of 14 (required age to be allowed to respond to such a survey without consent of a parent or legal guardian in Germany), live in Germany, and provide information on which federal state they live in. These criteria resulted in a usable sample of 4157 completed surveys. One thousand seven hundred sixty-nine of the participants in this sample were female, 1804 male (19 other, 565 missing values), with a mean age of 40.2 years (SD = 13.9). The proportion of subscribers to public transport tickets was rather high, which might be attributed to the fact that more than 60% of all participants lived in cities with more than 100,000 inhabitants that provide a good public transport system. Additional information on the sample can be found in Table 2.

**Table 2:** Sociodemographic characteristics and other transport related variables of the survey participants (*n* = 4157) in percent

Sociodemographic characteristic	Definition	In lockdown (*n* = 1645)	No lockdown (*n* = 2512)	All (*n* = 4157)
Gender	Female	44.9	41.0	42.6
	Male	42.4	44.1	43.4
	Not specified	0.4	0.5	0.5
	Not answered	12.3	14.5	13.6
Age	14–18	1.9	0.5	1.1
	19–30	32.4	28.9	30.3
	31–50	47.0	37.5	41.3
	51–65	16.4	28.6	23.8
	65+	2.3	4.5	3.6
	Not answered	0	0	0
Size of place of residence	Rural area *(small village; < 5000 inhabitants)*	7.5	12.7	10.6
	Small town *(5000–20,000 inhabitants*	6.3	10.4	8.8
	Mid-sized city *(20,000–100,000 inhabitants)*	6.6	23.8	17.0
	Major city *(100,000–500,000 inhabitants)*	24.9	31.8	29.1
	Small metropolis *(500,000–1 million inhabitants)*	22.2	14.1	17.3
	Big metropolis *(more than 1 million inhabitants)*	32.2	7.2	17.1
	Not answered	0.3	0.0	0.1
Subscription for public transport	Yes	50.8	41.8	45.3
	No, but I’d like to.	10.8	10.3	10.5
	No, I don’t need it.	37.5	47.0	43.3
	Not answered	0.9	0.9	0.9
Serviceable bicycles in the household	0	5.3	5.5	5.5
	1	15.3	14.3	14.7
	2	24.0	22.7	23.2
	3	16.9	17.6	17.3
	> 3	37.9	39.6	38.9
	Not answered	0.6	0.3	0.4
Cars in the household for sole or shared use	0	43.6	27.5	33.9
	1	39.9	43.2	41.9
	2	12.4	23.5	19.1
	3	2.1	3.7	3.1
	> 3	1.0	1.5	1.3
	Not answered	1.0	0.6	0.7

## Results

In the results section, we report findings on the full sample, and, when relevant, comparisons between federal states with and without lockdown. The questionnaire items used in the analysis are reported together with the respective results. Sample sizes are reported for each analysis. Variations in sample sizes are the result of missing values, i.e. participants choosing to not provide an answer to certain (subsets of) questions.

### Trips and trip purposes

Table 3 shows how often respondents made certain types of trips before the outbreak. Nearly 70% of the respondents reported to have travelled to work at least 4–5 times a week. More than 90% went out to make purchases at least once a week. Trips to leisure destinations were frequent as well. In contrast, only a small proportion of respondents made regular trips to accompany others (e.g. for day care) or had to be mobile for business-related errands.

**Table 3:** Distribution of answers to the item “*How often did you usually travel before the outbreak of the coronavirus to the following destination/purposes*?” (*n* = 4157) in percent, highest frequency in bold

	(almost) never	Less frequently than monthly	1–3 times a month	1–3 times a week	4–5 times a week	(almost) daily	n/a
Work	7.3	0.6	1.7	11.2	**39.3**	32.6	7.3
Business errands	**26.9**	10.6	18.9	17.9	5.3	4.4	16.0
Education	**32.2**	1.3	1.7	5.4	7.6	4.6	47.2
Purchases	0.5	0.6	4.9	**63.6**	18.6	11.2	0.6
Accompany others	**42.2**	7.0	9.2	9.5	5.0	5.1	22.0
to get to leisure activities	1.7	2.3	17.5	**52.7**	16.8	7.6	1.4
As leisure activity in itself	3.6	6.9	33.2	**42.0**	7.4	5.0	1.9
Other purposes	5.2	0.6	1.5	1.6	0.6	0.6	**89.9**

Asked about changes in their trips since the outbreak, nearly all respondents reported that certain trip purposes played no or only a reduced role compared to the pre-outbreak situation.^2^ The most significant reduction was reported for recreational trips (excursions, visits, etc.), which affected the majority (87.2%) of respondents. More than two-thirds reported the elimination of trips as a result of working from home (68.8%) and the cancellation of appointments (67.8%).Half of the participants (51.1%) stated that the use of video conferencing and phone calls resulted in a reduction of trips. There were no differences between federal states with and without lockdown in the types of trips that were reduced (recreational trips: 86.5% vs. 87.7%, working from home: 69.6% vs. 68.1%, cancellation of appointments: 67.6% vs. 68.0%, video conferencing/phone calls: 50.8% vs. 51.3%).

### Modal split

**Fig. 1:**
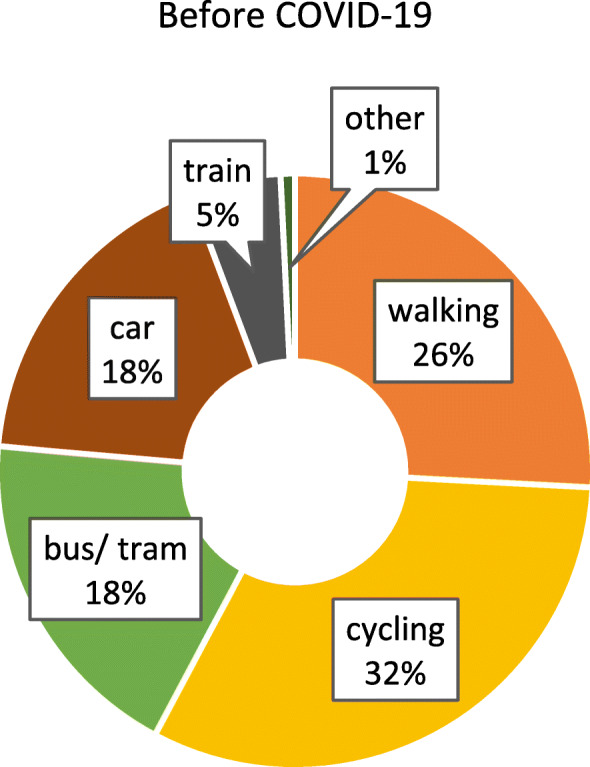
Modal split before the COVID-19 pandemic (*n* = 4100). Item: “*If your trips together add up to 100 percent, what proportion of these trips did the following means of transport usually have before the outbreak of coronavirus*?”

**Fig. 2:**
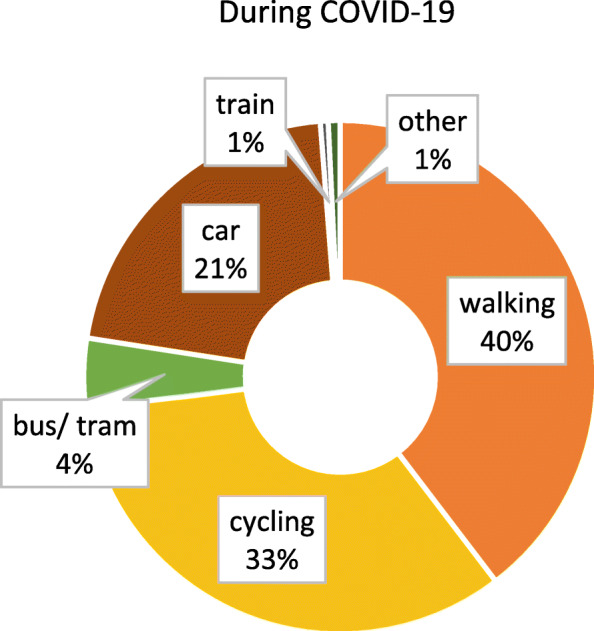
Modal split during the COVID-19 pandemic (*n* = 2407). Item *“If your trips together add up to 100 percent, what proportion of these trips did the following means of transport usually have since the outbreak of coronavirus?”*

Figure 1 shows the reported modal split before the COVID-19 outbreak. As can be seen, walking, cycling and short-distance public transport (bus/ tram) account for the largest shares (together 76%). Compared to the modal split in Germany as collected through representative surveys [14], the use of bicycles and public transport in our sample is rather high, while car usage is low. When looking at the modal split after the outbreak (Fig. 2), it becomes apparent that the proportion of trips made with public transport shrunk substantially, while the relative importance of walking, cycling and driving increased.

The applied chi-square test also showed that there is a significant difference between the modal split distribution before and during the COVID-19 outbreak, *χ*^*2*^(5) = 423.61, *p* < .000, *V* = 0.26.

**Fig. 3:**
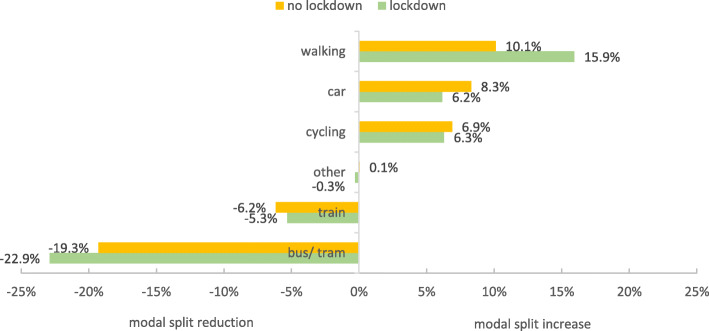
Modal split differences (during-before COVID-19) for the lockdown (*n* = 1023) and no lockdown group (*n* = 1368)

In order to examine differences between lockdown and no lockdown with respect to the modal split, the change of modal split was considered as difference of the shares during and before COVID-19 per means of transport for each group. As can be seen in Fig. 3, a similar pattern emerged for both groups: increases for walking, cycling and driving and decreases for public transport usage.

Comparing the two groups, it is noticeable that in the lockdown group there is a larger increase in walking (*U* = 602,095.50, *Z* = − 5.85, *p* < .001, *d* = 0.24) and a larger decrease in the use of public transport (*U* = 621,497.50, *Z* = − 4.71, *p* < .001, *d* = 0.19). Without lockdown a greater increase in car use was reported than with lockdown (*U* = 643,187.00, *Z* = − 3.43, *p* = .001, *d* = 0.14). For cycling, there was a larger increase among respondents who were not in lockdown. However, this difference is not statistically significant, *U* = 679,207.00, *Z* = − 1.23, *p* = .218, *d* = 0.05. A similar picture is found for long-distance public transport (train), where a larger decrease in the no lockdown group is shown. This difference is also not statistically verifiable, *U* = 670,822.50, *Z* = − 1.90, *p* = .058, *d* = 0.07.

### Specific changes in modal choice

**Fig. 4:**
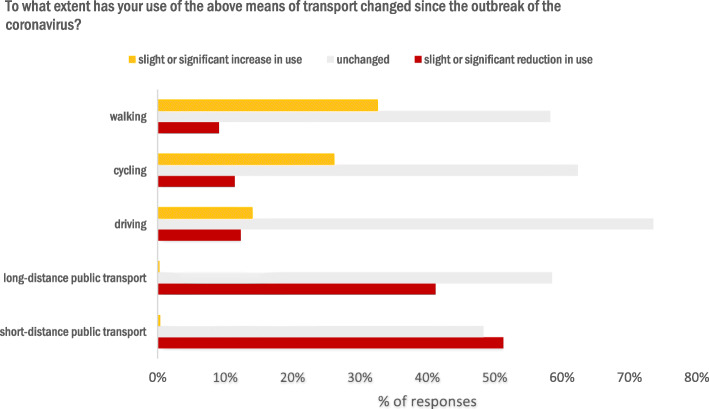
Reported changes in use of different means of transport (*n* = 4157). Item “*To what extent has your use of the above means of transport changed since the outbreak of the coronavirus*?”

More than half (58.8%) of the respondents confirmed that their use of different modes of transport had changed since the outbreak of the pandemic.^3^ Figure 4 gives an overview of how the use of different means of transport had changed. More than half of the participants reported to use short-distance public transport (tram, suburban and underground railway, bus) slightly or significantly less. Similar, although slightly less extreme than the latter, were the changes in the use of long-distance public transport (train rides). It should also be noted that the modal share of long-distance public transport was only 5% before the crisis, which might explain a considerable portion of the “no change” responses (i.e. not used before and not used during the crisis).

Walking and cycling showed changes in the opposite direction. About one third of those surveyed reported that they had increased walking since the outbreak of the virus, while about a quarter reported an increase in cycling. For driving (as a driver), the picture was different, with about three quarters of the respondents stating that their car use had not changed, while the remaining respondents were more or less evenly split into those who reduced and those who increased their driving.

When split into federal states with and without lockdown, we found slight differences in the reported change in transport use, whereby the effects are small. For each mode of transport, a comparison was made as to whether the response frequencies (in the categories: little or significantly more, unchanged and little or significantly less) differ between respondents with and without lockdown. Table 4 shows how respondents from states with and without lockdown adjusted their use of different modes of transport during COVID-19.

**Table 4:** Distribution of answers to the item *“To what extent has your use of the above means of transport changed since the outbreak of the coronavirus?”* in percent

mode of transport	response options	No lockdown (*n* = 2488 -2506)	In lockdown (*n* = 1634 -1643)	All (*n* = 4122 -4149)
Walking	use a little or significantly less	9,1	9,1	9,1
	use unchanged	62,1	52,4	58,3
	use a little or significantly more	28,9	38,5	32,7
Cycling	use a little or significantly less	10,9	12,2	11,4
	use unchanged	64,8	58,7	62,4
	use a little or significantly more	24,3	29,1	26,2
Driving	use a little or significantly less	14,4	9,2	12,3
	use unchanged	71,1	77,4	73,6
	use a little or significantly more	14,5	13,4	14,1
Short-distance public transport	use a little or significantly less	46,9	58,0	51,3
	use unchanged	52,6	41,9	48,3
	use a little or significantly more	0,5	0,2	0,4
Long-distance public transport	use a little or significantly less	38,3	45,7	41,2
	use unchanged	61,5	54,0	58,5
	use a little or significantly more	0,2	0,3	0,2

For walking, 10% more respondents without lockdown indicated no change compared to the group with lockdown. Also 10% more participants in lockdown reported walking a little or significantly more than participants without lockdown, χ^2^(2) = 44.10, *p* < .001, V = 0.10. The proportions of people who reported walking a little or significantly less did not differ between lockdown and no lockdown. With regard to cycling, there were differences in the answer categories between the groups, χ^2^(2) = 16.06, *p* < .001, V = 0.06. Respondents in the lockdown stated more often that they now used the bicycle slightly or significantly more than respondents without lockdown. The proportion of respondents who indicated that they used the bicycle slightly or significantly less is about the same between the groups. Participants without lockdown stated more often that their bicycle use remained unchanged than respondents in lockdown. For the comparison of the answer categories for car use, the groups also differed statistically significant, χ^2^(2) = 28.28, *p* < .001, V = 0.08. People in lockdown stated more often than people without lockdown that they had not changed their car use. Respondents without lockdown stated more often that they used the car a little or significantly less than people in lockdown. At the same time, respondents without lockdown also stated more often that they used the car more than people in lockdown. Differences between the groups could also be found for short-distance public transport, χ^2^(2) = 49.54, *p* < .001, V = 0.11. When using public transport, less use was more frequently reported by respondents in lockdown than by respondents without lockdown. In Addition, respondents in lockdown stated less frequently that their use of short-distance public transport had not changed than respondents without lockdown. The percentage of respondents who indicated an increase in use was at a similarly low level for both groups. The same picture emerges for long-distance public transport. Again, there were differences between the groups for the three answer categories, χ^2^(2) = 23.10, *p* < .001, V = 0.08. Respondents in lockdown more often stated that they used long-distance public transport slightly or significantly less than those without lockdown. Again, fewer respondents in lockdown stated that their use of long-distance public transport was unchanged compared to the group without lockdown. The frequencies in the answer category “I use slightly or significantly more” did not differ between groups for long-distance public transport.

**Fig. 5:**
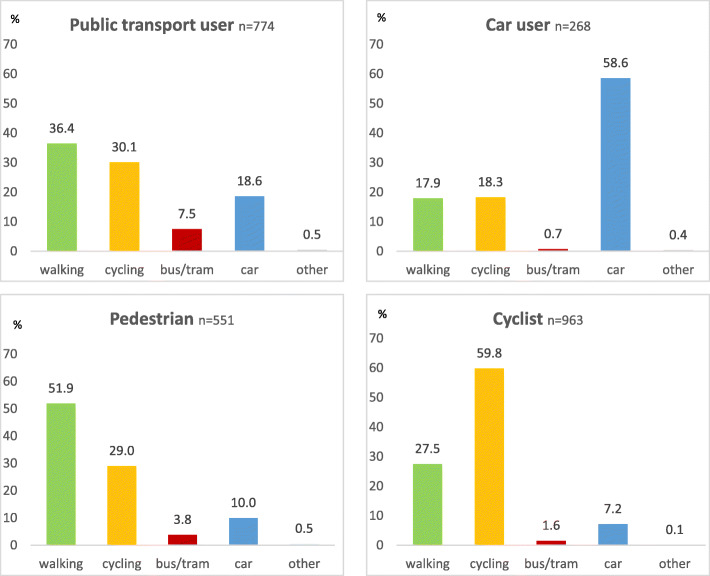
Reported main modes of transport during COVID-19 for respondents who identified as public transport users, car drivers, pedestrians or cyclists before COVID-19

To identify transitions from one mode to another, we split the survey participants in groups dependent on what type of transport usage they most identified with.^4^ For each of the identified subgroups, we then looked at their main mode of transport^5^ during the outbreak (see Fig. 5). Again, it becomes obvious that public transport was highly influenced by the crisis. Even those that identify primarily as users of public transport refrained from using this mode, with many of them walking, cycling, or, to a lesser degree, using the car instead. In contrast, among those that considered themselves primarily users of cars, pedestrians or cyclists, most stuck with their typical mode of choice.

Asked for an explanation of why a change in modal choice occurred with a predefined set of response options,^6^ many respondents agreed that the intention to reduce the risk of self-infection (72%) or the risk of infecting others (58%) were relevant factors. A few (23%) also agreed that their change in modal choice might have been due to the intent to strengthen their immune system. Seven percent of the participants changed their behaviour out of necessity, as their usual means of transport was not available.

### Urban and rural areas

**Fig. 6:**
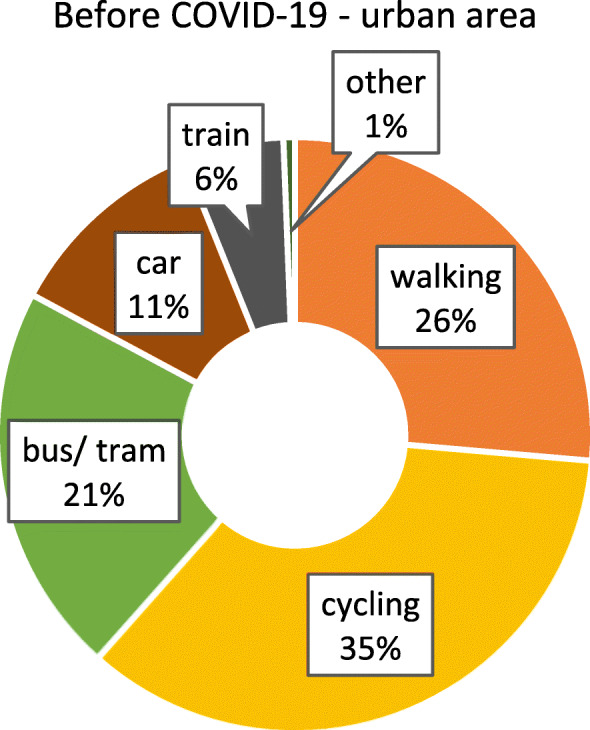
Modal split before the COVID-19 pandemic in urban areas (*n* = 2605). Item: “*If your trips together add up to 100 percent, what proportion of these trips did the following means of transport usually have before the outbreak of coronavirus*?”

**Fig. 7:**
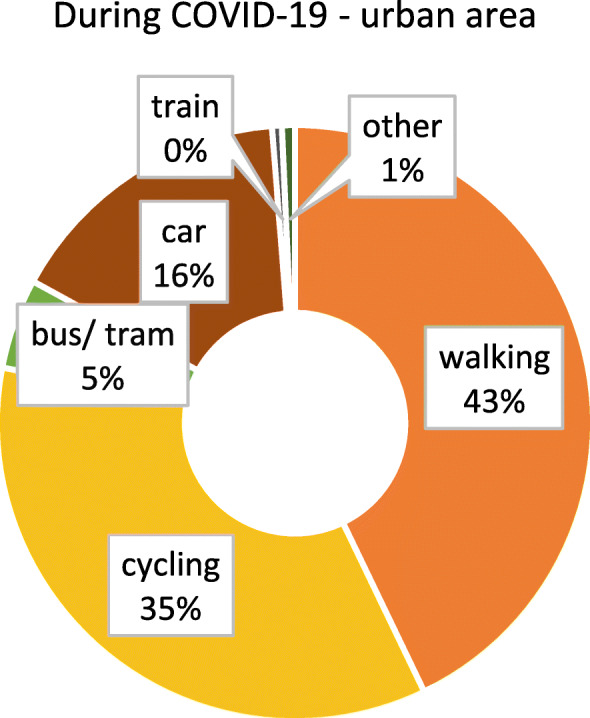
Modal split during the COVID-19 pandemic in urban areas (*n* = 1654). Item *“If your trips together add up to 100 percent, what proportion of these trips did the following means of transport usually have since the outbreak of coronavirus?”*

**Fig. 8:**
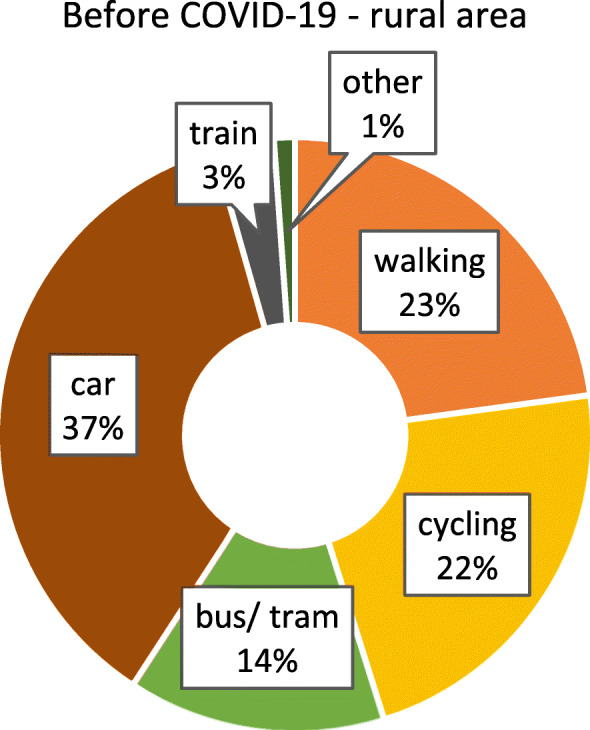
Modal split before the COVID-19 pandemic in rural areas (*n* = 791). Item: “*If your trips together add up to 100 percent, what proportion of these trips did the following means of transport usually have before the outbreak of coronavirus*?”

**Fig. 9:**
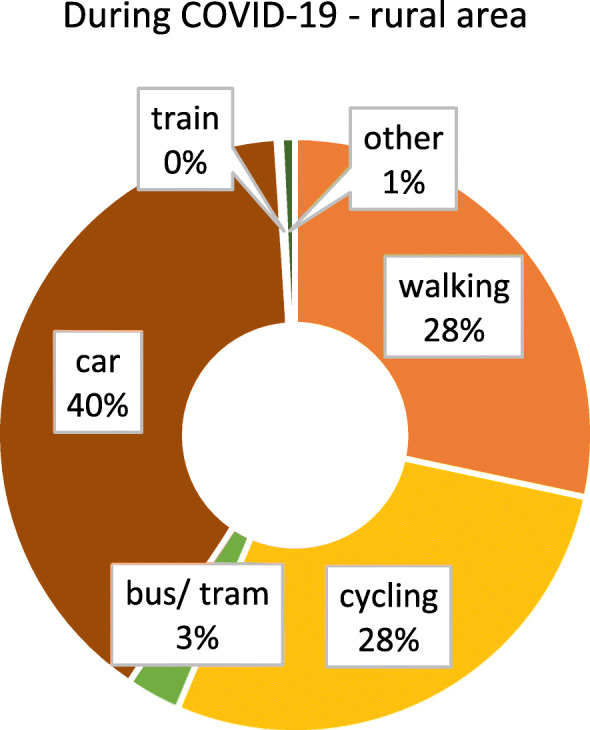
Modal split during the COVID-19 pandemic in rural areas (*n* = 394). Item *“If your trips together add up to 100 percent, what proportion of these trips did the following means of transport usually have since the outbreak of coronavirus?”*

As we were only able to identify minor, isolated differences between federal states with and without lockdown, we considered the possibility that other aspects, such as the degree of urbanisation, might impact on if and how people adjust their mobility behaviour. It can be suspected that highly urbanised regions, i.e. major cities, provide more opportunity to change behaviour, as the distances that would typically have to be covered are comparatively small, and the modes of transport available rather diverse. In contrast, in less urbanised / rural areas, behavioural options might be more limited. To assess this aspect, differences between respondents from highly urbanised (≥ 100.000 inhabitants) and somewhat rural (≤ 20.000 inhabitants, small towns included) areas were examined separately. Figures 6 and 7 show the modal split before and after COVID-19 for participants from urban areas. The patterns are similar to the results for the whole sample (irrespective of the size of place of residence, see section 3.2). Figures 8 and 9 show the modal split before and during COVID-19 for rural areas. As can be seen, the car usage in rural areas was comparatively high, while public transport and cycling played a smaller role than in urban areas. When looking at the differences in modal split before and during COVID-19, the pattern is somewhat similar to that for urban areas. The share of public transport decreased, whereas walking and cycling grew in importance. However, this increase in active mobility was not as extensive as in urban areas.

**Fig. 10:**
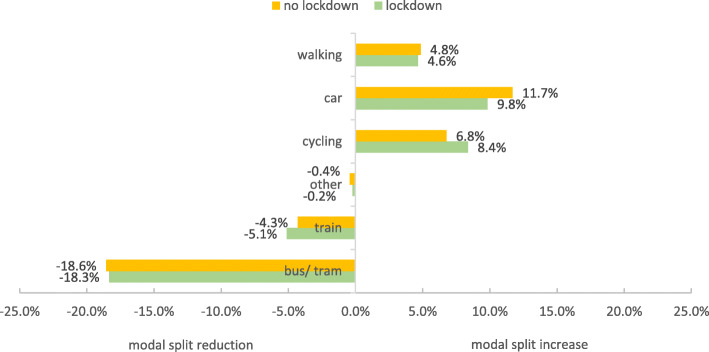
Modal split differences (during-before COVID-19) for rural areas for the lockdown (*n* = 119) and no lockdown group (*n* = 271)

Subsequently, the modal split differences (during-before COVID-19) between lockdown and no lockdown (Table 6) were examined. In principle, the findings for participants from urban areas are similar to the overall results reported in sections 3.2. This is plausible, as city dwellers account for around 63% of the total sample. The only thing noticeable about urban areas is that, in contrast to the analysis of overall differences in modal split changes (see 3.2), there is a larger decrease for long-distance public transport (train) without lockdown. Noteworthy are the findings for rural areas, which differ from urban areas in that no differences between participants with and without lockdown were found (see Fig. 10), indicating that the measure of lockdown had little impact on transport use in rural areas. There are tendencies that the modal share for driving without lockdown and for cycling in lockdown has increased during COVID-19. However, the lockdown and no lockdown group are not significantly different (see Table 6).

### Potential long term effects

Asked if they intended to maintain any behavioural changes^7^ after the crisis, 26% of the respondents indicated that they might do so, with 22% being undecided (*n* = 2108). While the majority of the public transport users surveyed who were using a different mode of transport (*n* = 712) stated that they intend to use bus and train again^8^ after the crisis (63%), about 20% expected to avoid public transport for a while even after the crisis is over.

## Discussion and conclusion

Aim of the analyses presented in this paper was to give some insight into Germans’ mobility behaviour in the early stages of the COVID-19 pandemic. As almost all who participated in our study confirmed, the crisis had a profound impact on their mobility behaviour, with typical trips that would occur under normal circumstances reduced or given up completely. The modal split saw a shift away from public transport, with increases both for forms of active mobility and travelling by car. This was also visible for both urban and rural areas. When looking at the individual level, it emerged that regular users of public transport often became pedestrians or cyclists, but also increasingly relied on the car, while users of other modes of transport mostly stuck with their usual choice. The risk of infecting oneself or others was frequently cited as the reason for a modal shift.

While the effects of the pandemic (and the corresponding countermeasures) overall were clearly visible, the role of the lockdown was less clear, with only isolated differences between states with and without lockdown, and only small effect sizes. It seems that, even if the lockdown had some minor effects, its role in the observed behavioural changes was minimal. It should be pointed out, however, that, in addition to the question of whether or not a lockdown was instated, the federal states’ response also differed with regard to when other restrictions were introduced (e.g. wearing masks on public transport or when inside stores, closure of restaurants and shops). The different timing of the introduction of restrictions might have functioned as a confounding factor. However, since these other restrictions hardly differed between the federal states during the period under review [25], there is no indication that the effect of the lockdown was masked by them. The overall risk perception of the health threat in everyday life may have played, similarly to other health threatening events from past, an important role in the considerations regarding mode choice.

When analysed separately, urban areas and rural areas showed somewhat similar patterns of change in modal split. Walking and cycling grew in importance, while public transport decreased, although in rural areas not as extensively as in urban areas. Differences in these changes (before vs. during COVID-19) between states with and without lockdown were only observed for urban areas, whereas the lockdown restriction seemed to have little impact on mobility behaviour in rural areas.

In the context of transport related emissions and their negative consequences for climate and health, and the associated push towards sustainable mobility, some of the behavioural adaptations we saw might be considered desirable, while others might be seen as problematic. The reduction in public transport use, while understandable, must be addressed to avoid any negative long-term effects. While, in our sample, we did not see a clear indication of increased car use in absolute terms, the reported relative increase is worrisome. To somewhat mitigate the effects, transport providers offered discounts on their tickets or extended their areas of application, especially for subscription card holders (e.g. [27]). In addition, to address potential health concerns and (re) build trust, buses, underground trains and trams were cleaned and disinfected at short intervals. For forms of active mobility, this extremely disruptive event can be seen as catalyst. Measures to facilitate walking and cycling during the pandemic were introduced within a short time (see [5] for an overview), among them pop-up bike lanes or adjustments in traffic signal timing.

However, especially with regard to public transport and bicycle use, it has to be acknowledged that the acquired sample is not completely representative for the German population as a whole. This might to some extend be the result of our sampling strategy, which made use of an existing mailing list that might have tilted slightly towards respondents from highly urbanised areas. As, not surprisingly, public transport suffered substantially during the crisis, the fact that we oversampled regular users of public transport might have resulted in an exaggeration of the overall impact of the pandemic on mobility behaviour. Also, the high number of frequent cyclists in the sample might have played a role in how modal choice shifted as well, in multiple ways. It might be argued that, with an already high level of bike ridership, the room for growth was limited as compared to a sample with a lower proportion of cyclists. At the same time, it can be suspected that it is easier for frequent (and therefore experienced, and presumably fit) cyclists to switch to the bike for certain trips, while, for an infrequent (or non-) user, it might appear more difficult to substitute, e.g., public transport with a bicycle.

Furthermore, at this stage, it is of course unclear if any of the reported short-term adaptations in behaviour will translate into more permanent changes. At the time of this paper’s writing, it was impossible to predict how or when the pandemic would end, so it is a matter of speculation if transport use and modal share would revert back to pre-COVID-19 levels or not. Follow up studies during the (still ongoing) pandemic, as well as in its aftermath, are required to answer that question. However, in light of the aspired transition towards a sustainable transport system, measures that might help perpetuate desired adaptations should be employed whenever possible, to make good use of this “window of opportunity” [22] that this crisis provided.

## Data Availability

The datasets used and analysed during the current study are available from the corresponding author on reasonable request.

## Appendix

**Table 5:** Distribution of responses per federal state in percent

Federal state	Total sample (*n* = 4157)
Baden-Württemberg	9,5
Bavaria	6,4
Berlin	11
Brandenburg	4,7
Bremen	1,5
Hamburg	3,2
Hessen	5,4
Mecklenburg-Vorpommern	3,2
Lower Saxony	11,7
North Rhine-Westphalia	21,7
Rhineland-Palatinate	1,3
Saarland	0,4
Saxony	15,9
Saxony-Anhalt	1,1
Schleswig-Holstein	1,7
Thuringia	1,3

**Table 6:** Differences between lockdown and no lockdown groups regarding modal split changes (during-before COVID-19) for rural and urban areas. *Note.* * *p* < .05, ***p* < .001

Rural areas	In lockdown (n = 119)	No lockdown (n = 271)	
	**Mean rank**	**Mean rank**	**Z-value**
Walking	190,31	197,78	−.604
Cycling	191,67	197,18	−.447
Driving	187,34	199,08	−.948
Short-distance public transport	199,11	193,91	−.426
Long-distance public transport	195,92	195,31	−.057
Others	198,98	193,97	−.825
Urban areas	In lockdown (*n* = 830)	No lockdown (*n* = 811)	
	**Mean rank**	**Mean rank**	**Z-value**
Walking	772,89	868,01	−4.069**
Cycling	810,09	832,17	−.946
Driving	797,43	844,08	−2.087*
Short-distance public transport	779,40	863,58	−3.616**
Long-distance public transport	847,69	793,69	−2.514*
Others	805,84	836,52	−3.051*

## Footnotes

1 German federal states differ in aspects such as size, population and population density. However, these differences are rather small when compared to, e.g., the federal structure of the US. The same is true for aspects such as the structure of the states’ economy. With regard to the analyses presented in this paper, we can assume that in most relevant variables, such minor differences between states are negligible.

2 Item: *How have your daily routes changed since the coronavirus outbreak?* Multiple selection possible for the options: elimination of trips through home office, elimination of trips through video conference/telephone calls, elimination of trips through the cancellation of appointments, elimination of trips due to day care/school closures, decrease of trips for purchases, increase of trips for purchases, elimination of trips for leisure purposes, no changes/adjustments, other.

3 Item: *Since the outbreak of the coronavirus, have you changed your transport use?* [Yes/No]

4 The item was introduced with the following sentence: “*Please tell us how you identify based on your means of transport.*”.

5 Item “*What is your main mode of transport you usually use for your trips since the outbreak of the coronavirus?”* Response options: walking, cycling, bus/train/tram (short-distance public transport), driving (car), motorbike/−scooter (PTW), e-scooter, train (long-distance public transport), I combine several means of transport.

6 Item „*For what reasons have you increasingly used another means of transport in recent days?*” (*n* = 2445). Response options (multiple selection): usual means of transport not available, reducing the risk of infection to self, reducing the risk of infection to others, strengthening the immune system, avoiding congestion caused by increased car use, other (commentary field).

7 Item „*I will maintain the changes in my traffic behaviour even after the corona crisis.*“Response options: reject completely, rather decline, partly, rather agree, agree completely, n/a

8 Item „*After the corona crisis I will avoid public transport (bus and train) for a while”.* Response options: reject completely, rather decline, partly, rather agree, agree completely, n/a
